# Current Drugs to Treat Infections with Herpes Simplex Viruses-1 and -2

**DOI:** 10.3390/v13071228

**Published:** 2021-06-25

**Authors:** Lauren A. Sadowski, Rista Upadhyay, Zachary W. Greeley, Barry J. Margulies

**Affiliations:** 1 Towson University Herpes Virus Lab, Department of Biological Sciences, Towson University, Towson, MD 21252, USA; lsadow2@students.towson.edu (L.A.S.); rupadh1@students.towson.edu (R.U.); zgreel1@gmail.com (Z.W.G.); 2 Towson University Department of Chemistry, Towson, MD 21252, USA; 3 Molecular Biology, Biochemistry, and Bioinformatics Program, Towson University, Towson, MD 21252, USA

**Keywords:** acyclovir, ganciclovir, cidofovir, vidarabine, foscarnet, amenamevir, docosanol, nelfinavir, HSV-1, HSV-2

## Abstract

Herpes simplex viruses-1 and -2 (HSV-1 and -2) are two of the three human alphaherpesviruses that cause infections worldwide. Since both viruses can be acquired in the absence of visible signs and symptoms, yet still result in lifelong infection, it is imperative that we provide interventions to keep them at bay, especially in immunocompromised patients. While numerous experimental vaccines are under consideration, current intervention consists solely of antiviral chemotherapeutic agents. This review explores all of the clinically approved drugs used to prevent the worst sequelae of recurrent outbreaks by these viruses.

## 1. Introduction

The world of anti-herpes simplex (anti-HSV) agents took flight in 1962 with the FDA approval of idoxuridine [1,2]. Since then, advances in understanding the genetics of herpes simplex viruses-1 and -2 (HSV-1 and -2) and their enzymology have opened the doors to many new, approved, and active pharmaceuticals, all provided as completely synthetic entities, to treat herpes simplex infections. 

## 2. HSV-1 and -2 Infection

The human herpesviruses HSV-1 and HSV-2 (HSVs) are major human pathogens in the simplexvirus family [3]. Both viruses infect people of all ages, with HSV-1 being more prevalent than HSV-2 [3]; the seroprevalence of the latter tends to increase in different populations as they age [4]. Once primary infection occurs, these viruses tend to retreat to local ganglia, where they remain latent for an indeterminant amount of time [3]. Nonetheless, at various times during the life of the host, the latent genomes of these viruses may reactivate and cause productive, lytic infections that may result in clinical signs and symptoms, such as skin lesions, genital sores, keratitis, whitlow, or other mucocutaneous pathologies [3]. In the most extreme cases, HSVs can cause fatal systemic infections or encephalitis, problems typically most associated with immune naïve or immunocompromised patients [3]. Therefore, providing antiviral intervention for those most severely affected by these viruses is necessary.

## 3. Nucleoside Analogs

The majority of anti-HSV drugs are nucleoside analogs that directly target the viral DNA polymerase when in their active form. The virally encoded DNA polymerase provides an essential function, and interfering with this enzyme results in inhibition of viral DNA replication, therefore preventing the production of infectious virions. Although this mechanism is effective, a potential issue is that these agents could also target the host DNA polymerase and lead to higher toxicity. All nucleoside analogs require tri-phosphorylation before binding to and inhibiting the viral DNA polymerase at its active site [5]. 

The first phosphorylation of antiviral nucleoside analogs typically occurs via a viral enzyme, thymidine kinase (TK); the subsequent di- and tri-phosphorylations are enacted through cellular kinases [6]. One benefit of this pathway is that host toxicity is limited because these drugs require the presence of said viral TK, which only appears during the early phase of an active, lytic HSV infection. Without the viral TK, these anti-HSV drugs tend not to become activated and therefore cannot inhibit any DNA polymerase. Nonetheless, the host’s own TK could potentially phosphorylate the drug first, but after cellular kinases tri-phosphorylate it, the drug will still have a higher affinity toward inhibiting the virus’ DNA synthesis over that of the host [7].

The main basis for resistance to nucleoside analogs resides in mutations in TK. Hence, some antiherpetic drugs that utilize a TK-directed pathway and may be less useful in the face of such resistance include acyclovir, valacyclovir, penciclovir, famciclovir, trifluridine, idoxuridine, vidarabine, sorivudine, brivudine, ganciclovir, and valganciclovir.

## 4. Acyclovir, the First in Its Class of Antiherpetic Drugs

**Figure 1:**
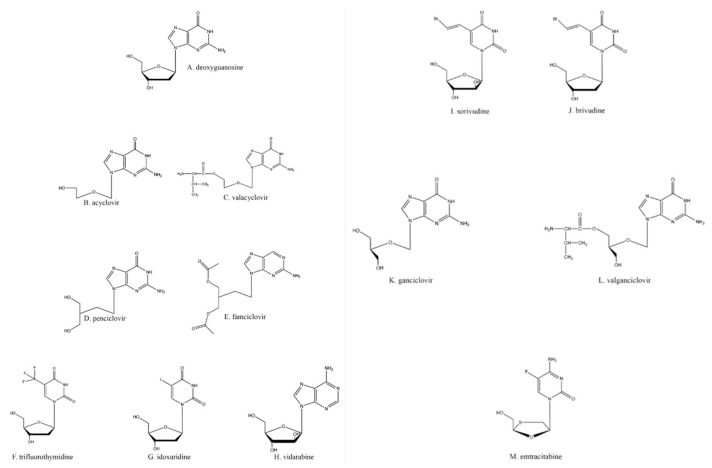
Nucleoside analogs.

Many of the antiherpetic nucleoside analogs primarily in clinical use are based on the core structure of deoxyguanosine (Figure 1A). Acyclovir (ACV; Figure 1B) is one of the most commonly used of these; it is activated by TK and inhibits both TK and DNA polymerase activities. ACV itself is a competitive inhibitor of the viral TK [8], whereas ACV-triphosphate (ACV-TP) acts as a competitive suicide inhibitor for the viral DNA polymerase [9]. Although ACV is known as a chain terminator, the nucleotide after ACV can be added to the growing DNA chain, but this forms a suicide inhibitor complex [10] in which the exonuclease activity of the DNA polymerase cannot excise ACV to restore activity; HSV DNA polymerase then gets caught in an ever-cycling trap of adding a few nucleotides, excising those nucleotides back towards the ACV, and repeating the cycle. Thus, the inactivation of the viral DNA polymerase prevents the complete replication of the viral genome and subsequently the formation of mature virions [11].

ACV toxicity in the host is generally low because of the requirement of viral TK for the first phosphorylation. Nonetheless, host TKs are able to perform the first phosphorylation of ACV at an extremely low level; typically, ACV-TP is found at a 40–100× greater concentration in infected cells than in uninfected cells [8], reflective of a measured selectivity index of 869 [12]. Furthermore, the toxicity of ACV is also abrogated because ACV-TP is a poor inhibitor of host DNA polymerase [13]. 

Viral resistance to ACV can be achieved through two different mechanisms: mutations to the DNA polymerase or mutations to the viral TK. Mutations to the DNA polymerase would disrupt the active site. However, the most common mutations that affect ACV-TP’s activity against viral DNA polymerase usually result in weak inhibition of DNA synthesis because they only slightly diminish the affinity for the ACV-TP in the active site [14]. More important are the more prevalent mutant TKs, which are not able to phosphorylate ACV. Strains of HSV that have ACV-resistant TKs lower drug activation and prevent the subsequent host enzyme phosphorylation of ACV [14,15]. Moreover, single and multiple mutations are more commonly found in the TK gene than in the DNA polymerase gene [14].

One other potentially problematic issue with ACV is its low oral bioavailability, which may be obviated by intravenous dosing [16,17]. This problem has been obviated with the introduction of valacyclovir (VaCV; Figure 1C), a prodrug of ACV created by the esterification of valine to ACV [18]; VaCV has a much higher absorption rate in the gut, resulting in less wasted drug with every oral dose [18]. As a prodrug, VaCV is converted through metabolism in the liver and kidney into ACV and valine by biphenyl hydrolase-like protein, which cleaves the esterified amino acid from the molecule [19,20]. Once VaCV is converted into ACV, all the properties and mechanisms of action as stated previously for ACV are the same.

## 5. Other Nucleoside Analogs

Another anti-HSV guanosine analog is penciclovir (PCV; Figure 1D). Although PCV and ACV are both analogs of the same nucleoside, the slight differences in structure between the drugs (Figure 1B,D) have led to minor differences in their pharmacology. PCV has about a 100× higher affinity for HSV TK compared to that of ACV [21]. This leads to much higher levels of PCV-TP than ACV-TP in vivo. Alternatively, ACV-TP shows about a 100× greater affinity for viral DNA polymerase than PCV-TP [21]. 

The differences between ACV’s and PCV’s molecular structures also result in PCV having oral absorption significantly lower than that of ACV; PCV’s oral bioavailability is 1.5%, and its in vivo half-life is 2–2.5 h [22,23,24]. As a potential solution to this issue, PCV has been acetylated, resulting in its prodrug famciclovir (FamCV; Figure 1E) [24], which has a much higher oral uptake (up to 73% of the dose) [25]. FamCV is rapidly converted to PCV by aldehyde oxidase in the liver, after which the PCV enters general circulation as the active drug [26]. VaCV and FamCV are some of the most widely available antivirals because of their high oral bioavailability and rapid metabolism into their active forms, although once converted into active drugs, their in vivo half-lives are not improved over those of the parent compounds.

Trifluridine (TFT; Figure 1F) and idoxuridine (IDU; Figure 1G), while still in use, are much less commonly employed antiherpetic drugs due to their toxicity. Both TFT and IDU are used optically to limit systemic toxicity [27], such as dermatitis and local burning [28] and bone marrow suppression [29]. TFT and IDU are both deoxyuridine analogs and utilize the same activation and antiviral schemes as the drugs previously described. 

Vidarabine (Figure 1H), a general polymerase inhibitor, was first synthesized as an anti-cancer drug [30]. The drug is an adenosine analog that retains activity against IDU- and ACV-resistant HSVs. However, because vidarabine acts indiscriminately on all polymerases, the drug can also impact host cell activities such as ribonucleoside reduction (by ribonucleoside reductase) and RNA polyadenylation [31]. Therefore, vidarabine suffers from much more limited clinical utility because of its mutagenic and oncogenic potential [32]. Hence, despite vidarabine being the first clinically approved antiviral drug, it is no longer available in the United States [33]. It should also be noted that vidarabine is less effective than other available antiherpetic drugs for treating HSV keratoconjunctivitis [34], so it suffers from poorer clinical reliability except in extreme cases.

Sorivudine (Figure 1I) and brivudine (Figure 1J) are thymidine analogs that inhibit DNA replication in the same fashion as described above. Brivudine ([(E)-5-(2-bromovinyl)-2′-deoxyuridine]) has greater potency than ACV against the varicella-zoster virus (VZV), another human alphaherpesvirus [35,36]; the drug appears to be not nearly as effective against HSV-1 because of the higher rates of HSVs that are resistant to these two medications [35,37]. 

Ganciclovir (GCV; Figure 1K), a guanosine analog like ACV, has an extra hydroxyl group on the ostensible 3′ carbon when compared to ACV. Ganciclovir is used primarily for cytomegalovirus (CMV) infections since most clinical CMV strains are still sensitive to it [38]. Although GCV’s anti-CMV activity can be attributed to its activation with the first phosphate being added via the UL97 kinase encoded by CMV [39], the HSV TK can also activate GCV via primary phosphorylation [40,41]. Similar to ACV, GCV exhibits poor oral bioavailability; subsequent work resulted in the creation of the VaCV analog valganciclovir (VGCV; Figure 1L) by esterifying valine to GCV [42]. As with VaCV, once inside the body, VGCV is converted into its active form, GCV [43].

## 6. Nucleotide Analogs: Cidofovir, Adefovir, and Brincidofovir

**Figure 2:**
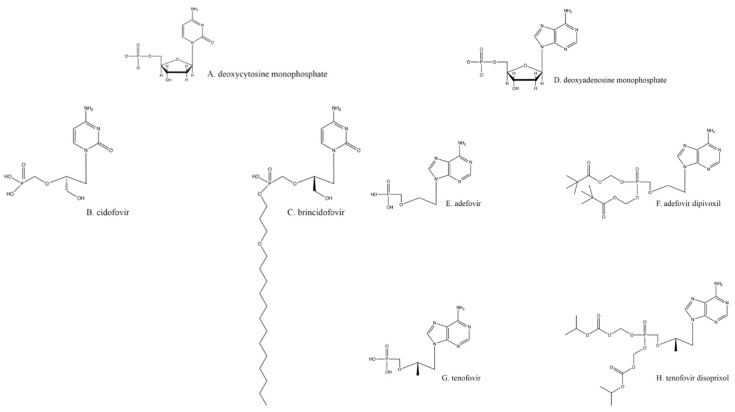
Nucleotide analogs.

Resistance to the drugs previously described is primarily acquired through mutations to the TK [44]. As previously noted, all the nucleoside analogs require TK for phosphorylation to initiate their antiviral activity, and TK mutants can no longer activate those drugs. Hence, without phosphorylation, none of the nucleoside analogs are active against herpesviruses. One way to overcome this requirement for TK is to use a nucleotide analog that already has a monophosphate attached, such as ones based on deoxycytosine monophosphate (Figure 2A), like cidofovir (CDV; Figure 2B). None of these nucleotide analogs require activation by TK.

CDV is di- and tri-phosphorylated by cellular kinases, much like nucleoside analogs [45]. The resulting cidofovir–triphosphate binds to the HSV DNA polymerase and is incorporated into the growing viral DNA chain, which reduces the speed of elongation. Furthermore, if two cidofovirs are incorporated adjacently in a growing HSV DNA chain, elongation is terminated [11]. Resistance to CDV occurs through mutations in the HSV DNA polymerase, typically with substitutions of amino acids that occur in less conserved regions of the enzyme [46,47]. Likewise, these mutations may allow the virus to become resistant to other drugs that are nucleoside analogs. A major drawback to the use of CDV is higher host toxicity, primarily in the kidneys [48].

Due to the limited bioavailability of CDV, the lipidated precursor brincidofovir (Figure 2C) has been created and is currently in clinical trials [49,50]. The increased biological distribution of the latter drug leads to greater virus inhibition at a lower overall dose [51]. The difference in brincidofovir metabolism also makes it a less toxic drug overall [48].

An analog of deoxyadenosine monophosphate (Figure 2D), adefovir (Figure 2E), is a drug already licensed as an anti-hepatitis B virus agent [52]. Adefovir also inhibits HSV replication [53,54]. Because of its similarity to CDV and tenofovir (Figure 2G), adefovir does not require activation by TK. Adefovir is primarily administered orally as a prodrug, adefovir dipivoxil (Figure 2F; [55]), with diesterified pivalic acids on its primary phosphate group [56]; this chemical alteration improves bioavailability, oral absorption, and other pharmacologic characteristics [56]. The pivalic acids adducts are removed through first-pass metabolism to create the active drug [56]. Adefovir treatment of HSV infections is not a common practice, partly because its use against any virus has been shown to cause nephrotoxicity [55,57] and partly because it can even help select for multidrug-resistant strains. Therefore, this option is best reserved for a minority of cases.

## 7. Non-Nucleoside/Nucleotide Inhibition of Herpes DNA Polymerase

**Figure 3:**
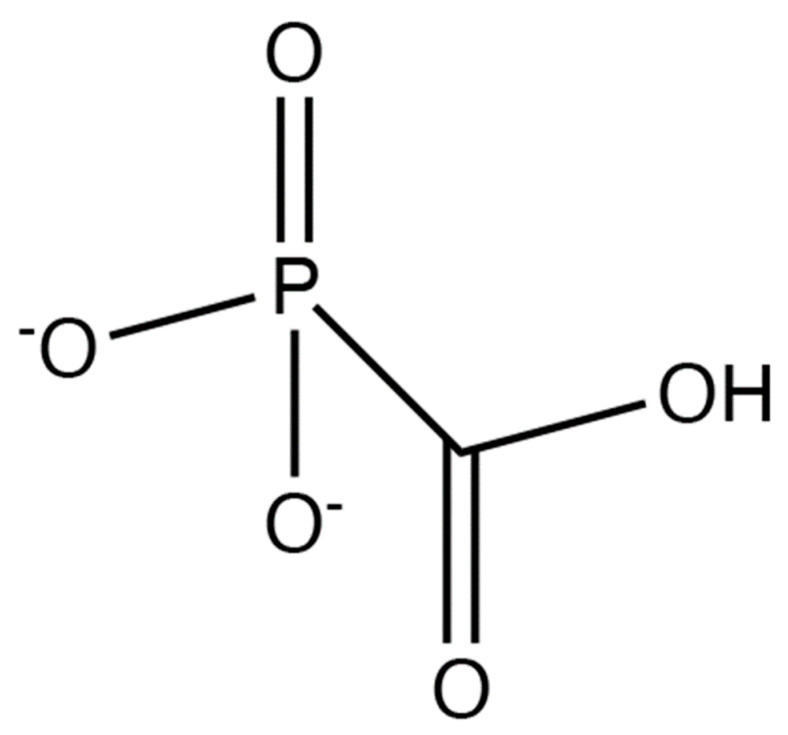
Foscarnet.

Foscarnet (Figure 3) is a pyrophosphate analog that reversibly binds to the viral DNA polymerase and hence does not require activation by TK. Unlike other antiherpetic drugs that bind to the viral DNA polymerase, the binding of foscarnet does not result in chain termination; the drug binds at the pyrophosphate binding site, within the active site of the herpesvirus DNA polymerase, preventing nucleotides from binding to the active site and from being incorporated into the growing DNA strand [58]. Foscarnet also differs from nucleoside-based drugs because it exists in its active form and requires no further modifications to inhibit herpesviruses. Since foscarnet does not require phosphorylation by the viral TK, it can also be used to inhibit ACV-resistant herpesviruses [46]. However, foscarnet is poorly selective. Since this drug does not get activated by viral enzymes and it binds to the active site of all polymerases, it has a higher potential to bind to and inhibit host DNA polymerases. While foscarnet shows 100× greater affinity for viral DNA polymerases than it does for human DNA polymerases [59], this level of difference may not be high enough for patients who are sensitive to the drug’s side effects, including acute nephrotoxicity [60,61], hypocalcemia [62], electrolyte disturbances, nausea, penile ulcerations, seizures, and metabolic disturbances [61,63]. On a cellular level, as the dosage of foscarnet increases, cell division slows by 50%; all phases of mitosis are impacted in some capacity as the G1, G2, and S phases are all greatly shortened [64]. Although foscarnet may inhibit host DNA replication, the greater inhibitory effects on viral DNA replication dictate that foscarnet can still be used therapeutically for ACV-resistant HSVs. Nonetheless, patients who have neurological or cardiovascular abnormalities while taking calcium-foscarnet must stop taking it [62]. Resistance to foscarnet typically appears in mutations at the pyrophosphate binding site of virus DNA polymerases.

## 8. Helicase/Primase Inhibitors

**Figure 4:**
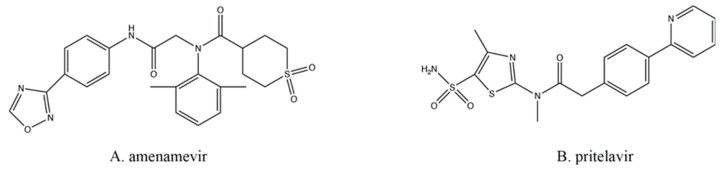
Helicase–primase inhibitors.

All the drugs discussed above typically target viral DNA replication at the elongation step, which means that individual mutations in only one gene (e.g., the TK or the DNA polymerase) could result in resistance to multiple drugs. Therefore, targeting other viral processes, at other loci, would prevent cross-resistance from appearing by such single mutations. One of these newer classes of antiherpetics is the helicase–primase inhibitors (HPIs; Figure 4), which also do not require preliminary phosphorylation by TK.

During replication of viral DNA, the helicase and primase enzymes (encoded by HSV-1 UL5 and UL52) form a complex that can separate the strands of DNA while also inserting primers [65]. Amenamevir (AMV; Figure 4A), approved for clinical use in Japan [66], and pritelivir (PTV; Figure 4B; still in clinical trials) are two of the most promising HPIs developed [67]. Both are active in their native state and require no modifications in order to inhibit the virus. Both drugs act similarly, likely by preventing the helicase–primase complex from forming by preventing the precise protein–protein interactions required between the UL5- and UL52-encoded proteins [67]. 

While it is not entirely understood at a molecular level exactly how the HPIs prevent the helicase–primase complex from forming, drug resistance appears through mutations to either the helicase, the primase, or both [68]. Most HPI resistance mutations already exist in the HSV population at a frequency of about 10^−6^ [69,70], and those mutants are not necessarily induced or selected by exposure to the inhibitors [68]. Nonetheless, these HPI-resistant mutants maintain wild-type levels of virulence in vivo [70].

## 9. Binding and Entry Inhibition

Other antiviral drugs act on the host cell to inhibit the virus [71]. The advantage to this approach is that resistance to the drug is less likely to appear, especially because these drugs are not subject to TK activation and ACV resistance mutations in that locus; while random mutations occur in both viruses and hosts, the mutation rate in viruses is much higher than it is in host cells [71,72,73,74]. On the other hand, targeting a host cell function may lead to higher toxicity, potentially limiting therapeutic use to only viral strains that are resistant to safer therapies.

**Figure 5:**
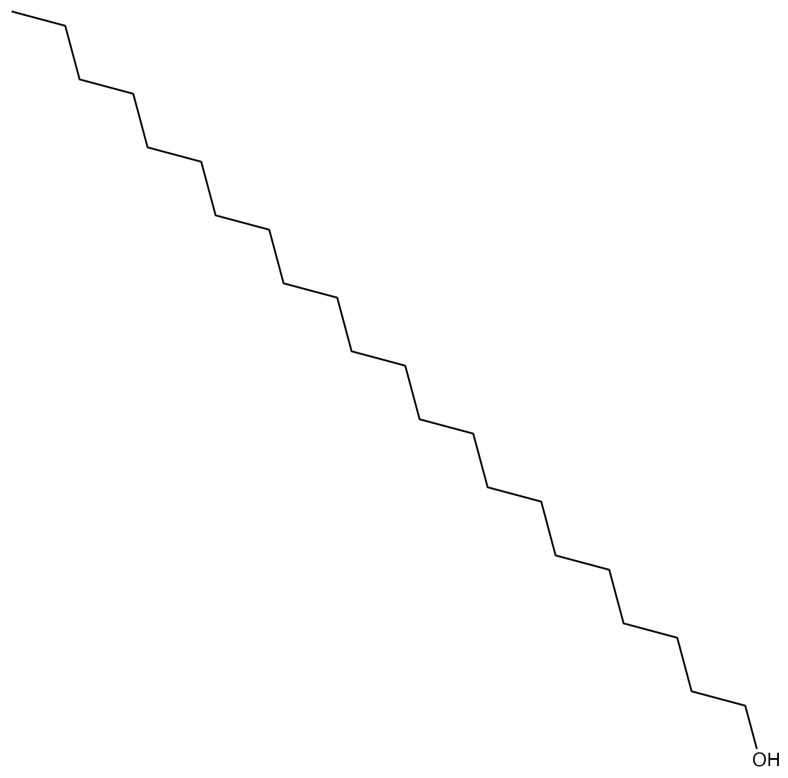
n-Docosanol.

*n*-Docosanol (Figure 5) is a long-chain, 22-carbon, primary alcohol offered over the counter. It likely inhibits a broad range of enveloped viruses that uncoat at the plasma membrane of target cells [75,76]. The drug appears to prevent binding and entry of HSVs by interfering directly with the cell surface phospholipids, which are required by the viruses for entry, and stabilizing them [76]. This activity tends to work well against ACV-resistant HSVs [75] and can even act synergistically with other anti-HSV drugs [77]. *n*-Docosanol is applied topically during prodrome to lessen the effect of a recurrent HSV outbreak; the drug even lessens the severity and duration of overt lesions. *n*-Docosanol is used only against labial, not genital, herpes outbreaks [78,79,80]. The parent compound itself is metabolized within cells primarily into phosphatidylethanolamine and phosphatidylcholine, typical cellular phospholipids, and it appears that these new derivatives of the drug exhibit the observed anti-HSV activity [76].

## 10. Licensed Drugs for Other Infectious Agents Can Also Inhibit Herpesviruses 

**Figure 6:**
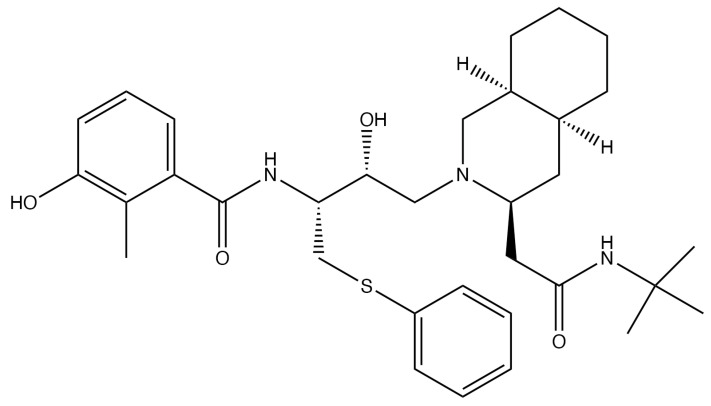
Nelfinavir.

Another drug that inhibits the virus by affecting the host cell is nelfinavir (NFV) (Figure 6), which also does not require activation by TK. Nelfinavir is a protease inhibitor used for HIV treatment that has curiously also been shown to inhibit HSV [5]. In the presence of NFV, the HSV capsids do not undergo secondary envelopment and therefore never reach maturity [5]. Even so, the mechanisms for this inhibitory effect are not well understood, although there is speculation that NFV interferes with intracellular membrane sorting and trafficking [48]. Since NFV has not been used clinically against HSVs, little is known about its overall safety and efficacy for such treatment schemes.

Emtricitabine (Figure 1M), a nucleoside analog typically used in pre-exposure prophylaxis (PrEP) schemes against HIV-1 in conjunction with the nucleotide analog tenofovir (Figure 2G; shown as its prodrug tenofovir disoproxil in Figure 2H) [81], has also found utility against HSV-2 [82,83]. Emtricitabine appears to be phosphorylated solely by cellular kinases [84]. Studies were originally designed to assess daily oral emtricitabine plus tenofovir (FTC/TDF) as a means to protect HIV-negative partners from acquiring that virus from HIV-positive partners [85]. As a side effect, it was shown that the same nucleoside/nucleotide analog regimen used to combat HIV-1 seroconversion could also reduce the risk of acquiring HSV-2 to 2.1 incidents per 100 person-years [86]; however, one study found that emtricitabine alone did not wholly lessen the chance of contracting HSV-2 [82]. Depending on the amount and means of drug delivery among other existing variables, FTC/TDF has been found to be effective at reducing the presence and severity of genital ulcers, a common HSV symptom [82]. 

## 11. Summary

While the arsenal of pharmacologic weapons we have to treat HSV infections is substantial, there is always room for improvement. To wit, IDU and TFT were supplanted by ACV and PCV, which have been further improved with VaCV and FamCV. The discovery of HPIs has revealed new HSV loci against which we can intervene, but it is likely that even these antiherpetics will be improved over the course of time. Moreover, time and effort in basic virology and pharmacology will inevitably lead to finding new molecular targets for intervention.

Regardless, none of these treatments are prophylactic, nor are they curative; these criteria may be overcome with an effective vaccine. Each antiviral agent mentioned above does not necessarily target or prevent primary infection. They also almost always require active replication, which means they cannot be used to rid the body of latent virus. Furthermore, as long as gene expression of latent virus is limited, the array of antiviral targets will similarly be constrained; more in-depth research must continue to be explored in the future to definitively aim at these much more complex problems.

## References

[1] Kaufman H., Martola E.L., Dohlman C. (1962). Use of 5-iodo-2′-deoxyuridine (IDU) in treatment of herpes simplex keratitis. Arch. Ophthalmol..

[2] Kaufman H.E. (1962). Clinical cure of herpes simplex keratitis by 5-iodo-2-deoxyuridine. Proc. Soc. Exp. Biol. Med..

[3] Roizman B., Knipe D.M., Whitley R.J., Knipe D.M., Howley P.M., Cohen J.I., Griffin D.E., Lamb R.A., Martin M.A., Racaniello V.R., Roizman B. (2013). Herpes Simplex Viruses. Fields Virology.

[4] Looker K.J., Garnett G.P. (2005). A systematic review of the epidemiology and interaction of herpes simplex virus types 1 and 2. Sex. Transm. Infect..

[5] Kalu N.N., Desai P.J., Shirley C.M., Gibson W., Dennis P.A., Ambinder R.F. (2014). Nelfinavir inhibits maturation and export of herpes simplex virus 1. J. Virol..

[6] Jia X., Schols D., Meier C. (2020). Lipophilic Triphosphate Prodrugs of Various Nucleoside Analogues. J. Med. Chem..

[7] Berdis A.J. (2008). DNA polymerases as therapeutic targets. Biochemistry.

[8] Elion G.B. (1982). Mechanism of action and selectivity of acyclovir. Am. J. Med..

[9] Furman P.A., St Clair M.H., Spector T. (1984). Acyclovir triphosphate is a suicide inactivator of the herpes simplex virus DNA polymerase. J. Biol. Chem..

[10] Yajima M., Yamada H., Takemoto M., Daikoku T., Yoshida Y., Long T., Okuda T., Shiraki K. (2017). Profile of anti-herpetic action of ASP2151 (amenamevir) as a helicase-primase inhibitor. Antivir. Res..

[11] Xiong X., Smith J.L., Chen M.S. (1997). Effect of incorporation of cidofovir into DNA by human cytomegalovirus DNA polymerase on DNA elongation. Antimicrob. Agents Chemother..

[12] Cunha A.C., Ferreira V.F., Vaz M.G.F., Cassaro R.A.A., Resende J., Sacramento C.Q., Costa J., Abrantes J.L., Souza T.M.L., Jordao A.K. (2020). Chemistry and anti-herpes simplex virus type 1 evaluation of 4-substituted-1H-1,2,3-triazole-nitroxyl-linked hybrids. Mol. Divers..

[13] McGuirt P.V., Furman P.A. (1982). Acyclovir inhibition of viral DNA chain elongation in herpes simplex virus-infected cells. Am. J. Med..

[14] Topalis D., Gillemot S., Snoeck R., Andrei G. (2016). Distribution and effects of amino acid changes in drug-resistant alpha and beta herpesviruses DNA polymerase. Nucleic Acids Res..

[15] Crumpacker C.S., Schnipper L.E., Chartrand P., Knopf K.W. (1982). Genetic mechanisms of resistance to acyclovir in herpes simplex virus. Am. J. Med..

[16] Van Dyke R.B., Connor J.D., Wyborny C., Hintz M., Keeney R.E. (1982). Pharmacokinetics of orally administered acyclovir in patients with herpes progenitalis. Am. J. Med..

[17] Gurgel Assis M.S., Fernandes Pedrosa T.C., de Moraes F.S., Caldeira T.G., Pereira G.R., de Souza J., Ruela A.L.M. (2021). Novel Insights to Enhance Therapeutics with Acyclovir in the Management of Herpes Simplex Encephalitis. J. Pharm. Sci..

[18] Birkmann A., Zimmermann H. (2016). HSV antivirals-current and future treatment options. Curr. Opin. Virol..

[19] Lai L., Xu Z., Zhou J., Lee K.D., Amidon G.L. (2008). Molecular basis of prodrug activation by human valacyclovirase, an alpha-amino acid ester hydrolase. J. Biol. Chem..

[20] Marsillach J., Suzuki S.M., Richter R.J., McDonald M.G., Rademacher P.M., MacCoss M.J., Hsieh E.J., Rettie A.E., Furlong C.E. (2014). Human valacyclovir hydrolase/biphenyl hydrolase-like protein is a highly efficient homocysteine thiolactonase. PLoS ONE.

[21] Earnshaw D.L., Bacon T.H., Darlison S.J., Edmonds K., Perkins R.M., Vere Hodge R.A. (1992). Mode of antiviral action of penciclovir in MRC-5 cells infected with herpes simplex virus type 1 (HSV-1), HSV-2, and varicella-zoster virus. Antimicrob. Agents Chemother..

[22] Boyd M.R., Bacon T.H., Sutton D. (1988). Antiherpesvirus activity of 9-(4-hydroxy-3-hydroxymethylbut-1-yl) guanine (BRL 39123) in animals. Antimicrob. Agents Chemother..

[23] Gill K.S., Wood M.J. (1996). The clinical pharmacokinetics of famciclovir. Clin. Pharm..

[24] Vere Hodge R.A., Sutton D., Boyd M.R., Harnden M.R., Jarvest R.L. (1989). Selection of an oral prodrug (BRL 42810; famciclovir) for the antiherpesvirus agent BRL 39123 [9-(4-hydroxy-3-hydroxymethylbut-l-yl)guanine; penciclovir]. Antimicrob. Agents Chemother..

[25] Filer C.W., Allen G.D., Brown T.A., Fowles S.E., Hollis F.J., Mort E.E., Prince W.T., Ramji J.V. (1994). Metabolic and pharmacokinetic studies following oral administration of 14C-famciclovir to healthy subjects. Xenobiotica.

[26] Clarke S.E., Harrell A.W., Chenery R.J. (1995). Role of aldehyde oxidase in the in vitro conversion of famciclovir to penciclovir in human liver. Drug Metab. Dispos..

[27] Agrahari V., Mandal A., Agrahari V., Trinh H.M., Joseph M., Ray A., Hadji H., Mitra R., Pal D., Mitra A.K. (2016). A comprehensive insight on ocular pharmacokinetics. Drug Deliv. Transl. Res..

[28] Silvestri D.L., Corey L., Holmes K.K. (1982). Ineffectiveness of topical idoxuridine in dimethyl sulfoxide for therapy for genital herpes. JAMA.

[29] Yamashita F., Komoto I., Oka H., Kuwata K., Takeuchi M., Nakagawa F., Yoshisue K., Chiba M. (2015). Exposure-dependent incorporation of trifluridine into DNA of tumors and white blood cells in tumor-bearing mouse. Cancer Chemother. Pharm..

[30] Sharma S., Mehndiratta S., Kumar S., Singh J., Bedi P.M., Nepali K. (2015). Purine Analogues as Kinase Inhibitors: A Review. Recent Pat. Anticancer Drug Discov..

[31] Whitley R.J., Tucker B.C., Kinkel A.W., Barton N.H., Pass R.F., Whelchel J.D., Cobbs C.G., Diethelm A.G., Buchanan R.A. (1980). Pharmacology, tolerance, and antiviral activity of vidarabine monophosphate in humans. Antimicrob. Agents Chemother..

[32] Aebersold P.M. (1979). Relative mutagenicity of nucleoside virostatic drugs in Chinese hamster ovary cells. Adv. Ophthalmol..

[33] Seley-Radtke K.L., Yates M.K. (2018). The evolution of nucleoside analogue antivirals: A review for chemists and non-chemists. Part 1: Early structural modifications to the nucleoside scaffold. Antivir. Res..

[34] Kaufman H.E. (1980). Antimetabolite drug therapy in herpes simplex. Ophthalmology.

[35] De Clercq E. (2004). Discovery and development of BVDU (brivudin) as a therapeutic for the treatment of herpes zoster. Biochem. Pharmacol..

[36] Wassilew S.W., Wutzler P., Brivddin Herpes Zoster Study Group (2003). Oral brivudin in comparison with acyclovir for herpes zoster: A survey study on postherpetic neuralgia. Antivir. Res..

[37] Andrei G., Balzarini J., Fiten P., De Clercq E., Opdenakker G., Snoeck R. (2005). Characterization of herpes simplex virus type 1 thymidine kinase mutants selected under a single round of high-dose brivudin. J. Virol..

[38] Noble S., Faulds D. (1998). Ganciclovir. An update of its use in the prevention of cytomegalovirus infection and disease in transplant recipients. Drugs.

[39] Gilbert C., Bestman-Smith J., Boivin G. (2002). Resistance of herpesviruses to antiviral drugs: Clinical impacts and molecular mechanisms. Drug Resist. Updates.

[40] Cheng Y.C., Grill S.P., Dutschman G.E., Nakayama K., Bastow K.F. (1983). Metabolism of 9-(1,3-dihydroxy-2-propoxymethyl)guanine, a new anti-herpes virus compound, in herpes simplex virus-infected cells. J. Biol. Chem..

[41] Cheng Y.C., Huang E.S., Lin J.C., Mar E.C., Pagano J.S., Dutschman G.E., Grill S.P. (1983). Unique spectrum of activity of 9-[(1,3-dihydroxy-2-propoxy)methyl]-guanine against herpesviruses in vitro and its mode of action against herpes simplex virus type 1. Proc. Natl. Acad. Sci. USA.

[42] Jung D., Dorr A. (1999). Single-dose pharmacokinetics of valganciclovir in HIV- and CMV-seropositive subjects. J. Clin. Pharmacol..

[43] Brown F., Banken L., Saywell K., Arum I. (1999). Pharmacokinetics of valganciclovir and ganciclovir following multiple oral dosages of valganciclovir in HIV-and CMV-seropositive volunteers. Clin. Pharm..

[44] Morfin F., Thouvenot D. (2003). Herpes simplex virus resistance to antiviral drugs. J. Clin. Virol..

[45] Bronson J.J., Ho H.T., De Boeck H., Woods K., Ghazzouli I., Martin J.C., Hitchcock M.J. (1990). Biochemical pharmacology of acyclic nucleotide analogues. Ann. N. Y. Acad Sci..

[46] Andrei G., Snoeck R., De Clercq E., Esnouf R., Fiten P., Opdenakker G. (2000). Resistance of herpes simplex virus type 1 against different phosphonylmethoxyalkyl derivatives of purines and pyrimidines due to specific mutations in the viral DNA polymerase gene. J. Gen. Virol..

[47] Razonable R.R. (2018). Drug-resistant cytomegalovirus: Clinical implications of specific mutations. Curr. Opin. Organ. Transpl..

[48] Meier P., Dautheville-Guibal S., Ronco P.M., Rossert J. (2002). Cidofovir-induced end-stage renal failure. Nephrol. Dial. Transpl..

[49] Ahmed A. (2011). Antiviral treatment of cytomegalovirus infection. Infect. Disord. Drug Targets.

[50] Marty F.M., Winston D.J., Chemaly R.F., Mullane K.M., Shore T.B., Papanicolaou G.A., Chittick G., Brundage T.M., Wilson C., Morrison M.E. (2019). A Randomized, Double-Blind, Placebo-Controlled Phase 3 Trial of Oral Brincidofovir for Cytomegalovirus Prophylaxis in Allogeneic Hematopoietic Cell Transplantation. Biol. Blood Marrow Transpl..

[51] Griffiths P., Lumley S. (2014). Cytomegalovirus. Curr. Opin. Infect. Dis..

[52] Balzarini J., Naesens L., De Clercq E. (1998). New antivirals-mechanism of action and resistance development. Curr. Opin. Microbiol..

[53] Aduma P., Connelly M.C., Srinivas R.V., Fridland A. (1995). Metabolic diversity and antiviral activities of acyclic nucleoside phosphonates. Mol. Pharmacol..

[54] Foster S.A., Cerny J., Cheng Y.C. (1991). Herpes simplex virus-specified DNA polymerase is the target for the antiviral action of 9-(2-phosphonylmethoxyethyl)adenine. J. Biol. Chem..

[55] Huang C., Yang X.H., Yang Y.L., Huang A.L., Shi X.F. (2018). Clinical-features analysis on 926 patients with virological breakthrough in chronic hepatitis B receiving nucleos(t)ide analogues. Eur. J. Intern. Med..

[56] Cundy K.C., Barditch-Crovo P., Walker R.E., Collier A.C., Ebeling D., Toole J., Jaffe H.S. (1995). Clinical pharmacokinetics of adefovir in human immunodeficiency virus type 1-infected patients. Antimicrob. Agents Chemother..

[57] Law S.T., Li K.K., Ho Y.Y. (2013). Acquired Fanconi syndrome associated with prolonged adefovir dipivoxil therapy in a chronic hepatitis B patient. Am. J. Ther..

[58] Vashishtha A.K., Kuchta R.D. (2016). Effects of Acyclovir, Foscarnet, and Ribonucleotides on Herpes Simplex Virus-1 DNA Polymerase: Mechanistic Insights and a Novel Mechanism for Preventing Stable Incorporation of Ribonucleotides into DNA. Biochemistry.

[59] Crumpacker C.S. (1992). Mechanism of action of foscarnet against viral polymerases. Am. J. Med..

[60] Leowattana W. (2019). Antiviral Drugs and Acute Kidney Injury (AKI). Infect. Disord. Drug Targets.

[61] Mareri A., Lasorella S., Iapadre G., Maresca M., Tambucci R., Nigro G. (2016). Anti-viral therapy for congenital cytomegalovirus infection: Pharmacokinetics, efficacy and side effects. J. Matern. Fetal Neonatal Med..

[62] Jacobson M.A., Gambertoglio J.G., Aweeka F.T., Causey D.M., Portale A.A. (1991). Foscarnet-induced hypocalcemia and effects of foscarnet on calcium metabolism. J. Clin. Endocrinol. Metab..

[63] Jayaweera D.T. (1997). Minimising the dosage-limiting toxicities of foscarnet induction therapy. Drug Saf..

[64] Stenberg K., Skog S., Tribukait B. (1985). Concentration-dependent effects of foscarnet on the cell cycle. Antimicrob. Agents Chemother..

[65] Matthews J.T., Terry B.J., Field A.K. (1993). The structure and function of the HSV DNA replication proteins: Defining novel antiviral targets. Antivir. Res..

[66] Shoji N., Tanese K., Sasaki A., Horiuchi T., Utsuno Y., Fukuda K., Hoshino Y., Noda S., Minami H., Asakura W. (2020). Pharmaceuticals and Medical Device Agency approval summary: Amenamevir for the treatment of herpes zoster. J. Dermatol..

[67] Shiraki K. (2018). Antiviral Drugs Against Alphaherpesvirus. Adv. Exp. Med. Biol..

[68] Field H.J., Biswas S. (2011). Antiviral drug resistance and helicase-primase inhibitors of herpes simplex virus. Drug Resist. Updates.

[69] Biswas S., Jennens L., Field H.J. (2007). Single amino acid substitutions in the HSV-1 helicase protein that confer resistance to the helicase-primase inhibitor BAY 57-1293 are associated with increased or decreased virus growth characteristics in tissue culture. Arch. Virol..

[70] Liuzzi M., Kibler P., Bousquet C., Harji F., Bolger G., Garneau M., Lapeyre N., McCollum R.S., Faucher A.M., Simoneau B. (2004). Isolation and characterization of herpes simplex virus type 1 resistant to aminothiazolylphenyl-based inhibitors of the viral helicase-primase. Antivir. Res..

[71] Lin K., Gallay P. (2013). Curing a viral infection by targeting the host: The example of cyclophilin inhibitors. Antivir. Res..

[72] Drake J.W., Hwang C.B. (2005). On the mutation rate of herpes simplex virus type 1. Genetics.

[73] Renner D.W., Szpara M.L. (2018). Impacts of Genome-Wide Analyses on Our Understanding of Human Herpesvirus Diversity and Evolution. J. Virol..

[74] Sanjuán R., Nebot M.R., Chirico N., Mansky L.M., Belshaw R. (2010). Viral mutation rates. J. Virol..

[75] Katz D.H., Marcelletti J.F., Khalil M.H., Pope L.E., Katz L.R. (1991). Antiviral activity of 1-docosanol, an inhibitor of lipid-enveloped viruses including herpes simplex. Proc. Natl. Acad. Sci. USA.

[76] Pope L.E., Marcelletti J.F., Katz L.R., Lin J.Y., Katz D.H., Parish M.L., Spear P.G. (1998). The anti-herpes simplex virus activity of n-docosanol includes inhibition of the viral entry process. Antivir. Res..

[77] Marcelletti J.F. (2002). Synergistic inhibition of herpesvirus replication by docosanol and antiviral nucleoside analogs. Antivir. Res..

[78] Woo S.B., Challacombe S.J. (2007). Management of recurrent oral herpes simplex infections. Oral Surg. Oral Med. Oral Pathol. Oral Radiol. Endodontol..

[79] Usatine R.P., Tinitigan R. (2010). Nongenital herpes simplex virus. Am. Fam. Phys..

[80] Leung D.T., Sacks S.L. (2004). Docosanol: A topical antiviral for herpes labialis. Expert Opin. Pharm..

[81] Deeks E.D. (2018). Bictegravir/Emtricitabine/Tenofovir Alafenamide: A Review in HIV-1 Infection. Drugs.

[82] Marcus J.L., Glidden D.V., McMahan V., Lama J.R., Mayer K.H., Liu A.Y., Montoya-Herrera O., Casapia M., Hoagland B., Grant R.M. (2014). Daily oral emtricitabine/tenofovir preexposure prophylaxis and herpes simplex virus type 2 among men who have sex with men. PLoS ONE.

[83] Celum C., Morrow R.A., Donnell D., Hong T., Hendrix C.W., Thomas K.K., Fife K.H., Nakku-Joloba E., Mujugira A., Baeten J.M. (2014). Daily oral tenofovir and emtricitabine-tenofovir preexposure prophylaxis reduces herpes simplex virus type 2 acquisition among heterosexual HIV-1-uninfected men and women: A subgroup analysis of a randomized trial. Ann. Intern. Med..

[84] Figueroa D.B., Madeen E.P., Tillotson J., Richardson P., Cottle L., McCauley M., Landovitz R.J., Andrade A., Hendrix C.W., Mayer K.H. (2018). Genetic Variation of the Kinases That Phosphorylate Tenofovir and Emtricitabine in Peripheral Blood Mononuclear Cells. AIDS Res. Hum. Retrovir..

[85] Baeten J.M., Donnell D., Mugo N.R., Ndase P., Thomas K.K., Campbell J.D., Wangisi J., Tappero J.W., Bukusi E.A., Cohen C.R. (2014). Single-agent tenofovir versus combination emtricitabine plus tenofovir for pre-exposure prophylaxis for HIV-1 acquisition: An update of data from a randomised, double-blind, phase 3 trial. Lancet Infect. Dis..

[86] Chaix M.L., Charreau I., Pintado C., Delaugerre C., Mahjoub N., Cotte L., Capitant C., Raffi F., Cua E., Pialoux G. (2018). Effect of On-Demand Oral Pre-exposure Prophylaxis With Tenofovir/Emtricitabine on Herpes Simplex Virus-1/2 Incidence Among Men Who Have Sex With Men: A Substudy of the ANRS IPERGAY Trial. Open Forum Infect. Dis..

